# *Curcuma longa* L. Rhizome Extract as a Poly(vinyl chloride)/Graphene Nanocomposite Green Modifier

**DOI:** 10.3390/molecules27228081

**Published:** 2022-11-21

**Authors:** Sławomir Wilczewski, Katarzyna Skórczewska, Jolanta Tomaszewska, Krzysztof Lewandowski, Waldemar Studziński, Magdalena Osial, Piotr Jenczyk, Hubert Grzywacz, Agata Domańska

**Affiliations:** 1 Faculty of Chemical Technology and Engineering, Bydgoszcz University of Science and Technology, Seminaryjna 3 Street, 85-326 Bydgoszcz, Poland; 2 Institute of Fundamental Technological Research, Polish Academy of Sciences, Pawinskiego 5B Street, 02-106 Warsaw, Poland; 3 Łukasiewicz Research Network—Institute for Engineering of Polymer Materials and Dyes, Marii Skłodowskiej-Curie 55 Street, 87-100 Toruń, Poland

**Keywords:** nanocomposites, graphene, poly(vinyl chloride), curcuma extract

## Abstract

In this work, a method to increase the dispersion of graphene (GN) in the matrix of rigid poly(vinyl chloride) (PVC) by using a natural plant extract from *Curcuma longa* L. (CE) is proposed. Currently, despite the increasing number of reports on the improvement of GN dispersion in PVC blends, still there is a need to find environmentally friendly and economical dispersion stabilizers. We proposed a stabilizer that can be easily obtained from a plant offering thermal stability and high effectiveness. PVC/GN nanocomposites stabilized with the proposed extract were investigated by SEM, AFM (structure), TGA, and Congo red test (thermal properties). Additionally, static and dynamic mechanical properties and electrical resistivity were measured. The use of CE as a graphene dispersant improved its dispersion in the PVC matrix, influenced tensile properties, increased the storage modulus and glass transition temperature, and extended the thermal stability time of nanocomposites. In this work, a CE extract is proposed as an efficient eco-friendly additive for the production of nanocomposites with an improved homogeneity of a nanofiller in the matrix and promising characteristics.

## 1. Introduction

Research concerning graphene application is carried out in many areas, such as medicine, chemistry, materials engineering, energetics, and electronics [1,2,3,4,5]. A large area of graphene-related research is its use as a filler for polymer nanocomposites; however, its use is limited by the lack of large-scale methods for obtaining a high-quality filler and its poor dispersion in the polymer matrix [6,7,8,9,10]. Despite numerous studies on polymer nanocomposites containing graphene, a relatively small number of them concern the modification of poly(vinyl chloride). This polymer is distinguished by high chemical resistance and favorable mechanical and performance characteristics [11,12,13]; however, it has low thermal stability under molten state processing conditions [14,15]. Therefore, it is widely modified with additives that improve its functionality [16,17,18,19,20,21]. Literature data on poly(vinyl chloride) nanocomposites with graphene fillers (PVC/GN) prove that a good dispersion of graphene in the matrix results in an increase in tensile strength, longitudinal modulus of elasticity [22,23,24,25,26,27], and impact resistance [27,28]. Such a composite is also characterized by higher thermal stability and glass transition temperature (T_g_) [24,25,26,27,29,30] as well as lower volume and surface resistivity in comparison with the matrix material [25,31,32].

The key problems to be solved in the case of PVC/GN nanocomposites, regardless of the method of their production, include obtaining a good dispersion of the filler in the matrix and increasing the filler–polymer interfacial interactions. Thus, a poor dispersion of the filler in the polymer matrix affects its physicochemical properties [23,24,25,26,27,28,30,31]. However, it should be emphasized that obtaining a good dispersion of the graphene filler in PVC is related to its low agglomeration and favorable interfacial interactions of the filler and polymer [22,24,26,28,31].

The tendency of graphene to form agglomerates results from strong π–π interactions and van der Waals forces [33,34,35,36]. The basic methods for increasing the dispersibility of GN are divided into noncovalent and covalent ones [2,33,35]. The covalent modification of graphene materials used in PVC composites is based on the use of chemical reagents that usually are harmful to health and the environment [37,38,39,40,41]. In addition, these methods change the structure of graphene, affecting its characteristics. Noncovalent methods are based on increasing the dispersion of graphene in the PVC matrix by its dispersion in a plasticizer [25] and mechanical exfoliation of graphite in the plasticizer environment [32]. Generally, they are based on the use of a modifier that significantly affects the properties of PVC [15]. 

An interesting method for noncovalent functionalization of graphene in order to limit its agglomeration is surface modification with curcumin (CU). The π–π interactions between CU aromatic rings and the graphene surface were used in its synthesis to reduce graphene oxide [42,43]. Curcumin was also used during the graphite exfoliation by sonication [44]. 

Curcumin is a natural, biologically active chemical compound belonging to the group of curcuminoids that are extracted from turmeric, particularly *Curcuma longa* L. The content of the curcuminoids in the plant depends on the environmental and soil conditions and ranges from 2% to 9% of curcuminoid content in the plant [45,46,47]. These compounds show high chemical stability in their natural form, which makes them ideal for many applications in engineering, medicine, and pharmacy [47,48]. In [44], the authors demonstrated that curcumin is thermally stable up to approximately 180 °C and in combination with graphene even up to approximately 220 °C. Some studies show that CU can be used as a polymer additive for its chemical stability [47]. It was used for plasticization, colorizing, and increasing the antibacterial resistance of PVC [49,50,51].

The aim of the presented study was to investigate the impact of the curcuminoid-based extract obtained from the rhizome of *Curcuma longa* L. on the structure and properties of PVC nanocomposites with graphene. The use of this extract as a component of the composite is a new, economical solution that is safe for health and the environment. Additionally, the use of CE improved the dispersion of graphene in PVC.

## 2. Results and Discussion

### 2.1. Analysis of Curcuma longa L. Rhizome Extract

**Figure 1:**
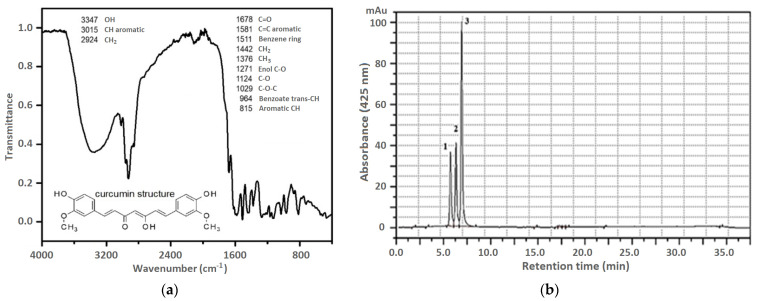
(**a**) FTIR spectra; (**b**) HPLC chromatogram of *Curcuma longa* L. rhizome extract.

Curcuminoids are the main active compounds contributing to the chemical activity of *Curcuma longa* L. Turmeric usually contains 2% to 9% of curcuminoids, mainly curcumin, approximately 77% [45,46,47]. The phenolic compounds are also the main active ingredients of turmeric alcoholic extracts. To confirm the presence of these compounds in the as-prepared extract, Fourier Transformed Infrared spectroscopy (FT-IR) was performed. Figure 1a shows the following bands, where the band at 3347 cm^−1^ can be attributed to the -O-H stretching vibration. The subsequent bands can be described as follows: 3015 cm^−1^ C-H aromatic stretching vibration, 2924 cm^−1^ -CH_2_ to asymmetric stretching, 1678 cm^−1^ C=O stretching, 1581 cm^−1^ C=C aromatic stretching, 1511 cm^−1^ benzene ring bending vibration, 1442 cm^−1^ CH_2_ bending, 1376 CH_3_ bending, 1271 cm^−1^ enol C-O peak, 1124 cm^−1^ C-O stretching, 1029 cm^−1^ C-O-C peak, 964 cm^−1^ benzoate trans-CH vibration, and 815 cm^−1^ aromatic CH bending. These bands are characteristic of curcumin, demethoxycurcumin, and bisdemethoxycurcumin functional groups [45,52,53].

In addition to curcuminoids, essential oils are the main active ingredients in turmeric. The analysis of the curcuminoids in turmeric is critical to determining the quality of the plant material or its processed products [54]. To determine the individual components of turmeric, HPLC and GC–MS were used in the paper. These methods were most often used and described in the literature [55]. The quantitative HPLC method has been validated. The three analytes showed good linearity (R^2^ ≥ 0.999) in the concentration range of 2–80 µg mL^−1^. The RSD values were 1.48%. The HPLC chromatogram of the fresh turmeric extract is shown in Figure 1b. Peaks 1, 2, and 3 showed similar UV–vis spectra that are characteristic of curcuminoids at wavelength *λ* = 425 nm. They were identified as bisdemetoxycurcumin (1), demethoxycurcumin (2), and curcumin (3), respectively, on the basis of the retention times compared with the retention times of the reference compounds.

It was calculated that the sum of the three curcuminoids’ content in the extract is 150 mg g^−1^, of which the curcumin content is 84 mg g^−1^. Based on the analysis, it can be concluded that curcumin was the most common compound, and the sum of the demethoxycurcumin and bisdemethoxycurcumin contents was lower than that of curcumin. Therefore, the turmeric extract can be classified as type A [56].

**Figure 2:**
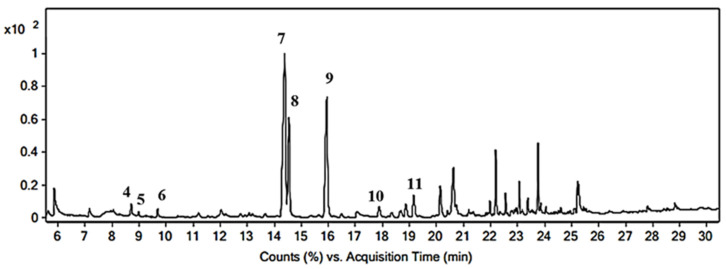
GC–MS chromatogram of *Curcuma longa* L. rhizome extract.

Volatile compounds in the turmeric extract were also identified from GC–MS analysis. In total, eight peaks were identified (Figure 2) when compared with the NIST database and literature data [56,57]. The following compounds have been identified: Ar-curcumene (4), (-)-zingiberene (5), *β*-sesquiphellandrene (6), Ar-turmerone (7), α-turmerone (8), β-turmerone (9), (6R,7R)-bisabolene (10), and (E)-atlantone (11). Mass spectra of the compounds are shown on Figures S1–S8 in Supplementary File.

Additionally, the ratio of the heights of individual compounds on the GC–MS chromatogram makes the extract qualify as type A. According to Xu et al. [56], the turmeric extract of type A is characterized by the fact that peak 7 is higher than peak 8, and peak heights are 5 and 6, which are in total less than 33% of the height of peak 4. This is exactly the case here. The analyses of the obtained extract from the rhizome of *Curcuma longa* L. confirm the effectiveness of the extraction. Curcuminoids have been shown to be the main active ingredients of CE used as an auxiliary in poly(vinyl chloride)/graphene nanocomposites.

### 2.2. Structure of PVC/GN Nanocomposites

**Figure 3:**
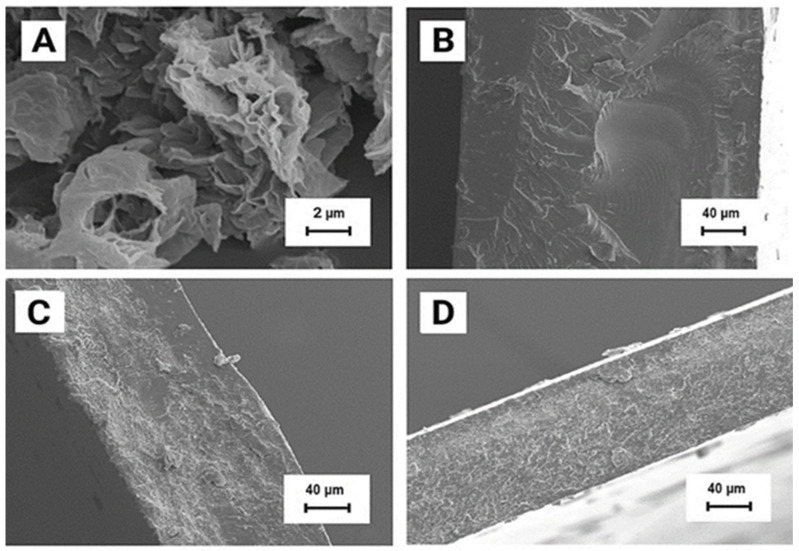
SEM images of (**A**) GN, (**B**) PVC, (**C**) PVC/1% GN, and (**D**) PVC/1% GN + CE.

The morphology of the nanocomposites’ cross section was investigated using scanning electron microscopy (SEM), where fracture was performed under liquid nitrogen conditions. Figure 3 presents unmodified ingredients and a graphene-filled PVC nanocomposite containing 1 wt.% of graphene. As can be seen in Figure 3A, graphene reveals multilayered wrinkle-like flake structures that are characteristic to the elasticity of the material and large size of graphene flakes [58]. The fracture area of poly(vinyl chloride) (Figure 3B) is characteristic of the brittle fracture of thermoplastics. Fractures of nanocomposites reveal a jagged morphology [27,28,31,59,60]. Based on SEM images of the fracture surfaces of nanocomposites (Figure 3C,D), GN dispersion in the materials was assessed. The image of a PVC/1% GN sample (Figure 3C) shows the presence of filler agglomerates in the fracture edge. On the other hand, the fracture area of the PVC/1% GN + CE nanocomposite (Figure 3D) is characterized by a homogeneous dispersion of the filler in the PVC matrix, and no areas of an unmodified polymer structure were observed.

**Figure 4:**
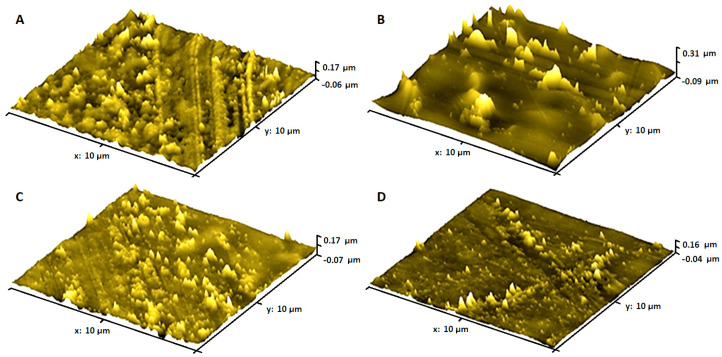
AFM 3D images of (**A**) PVC, (**B**) PVC/1% GN, (**C**) PVC + CE, and (**D**) PVC/1% GN + CE.

In order to assess the surface structure of the obtained composite films, atomic force microscopy was used, enabling the direct assessment of the dispersion of graphene materials in nanocomposites and the determination of their roughness [61,62]. Figure 4 presents 3D images of the surface structure of PVC, PVC + CE, and nanocomposites containing 1 wt.% of graphene. The test was carried out in contact mode on the surface that was not in contact with the Petri dish during the evaporation of the solvent (polymer film formation). Topography studies revealed the presence of corrugation and roughness (7.322 ± 0.424 nm) on the surface of unmodified poly(vinyl chloride), which results from solvent evaporation during the formation of the polymer film. The roughness was estimated to be 12.017 ± 0.302 nm for PVC + CE, 52.111 ± 1.257 nm for PVC/1% GN, and 36.216 ± 0.234 nm for PVC/1% GN + CE. A significant increase in the surface roughness of GN-containing nanocomposite films is the result of both solvent evaporation and the presence of a filler in the polymer matrix. The nanocomposites with the addition of CE had a lower roughness value than PVC/1% GN for the higher dispersion of the filler in the PVC matrix [63,64]. 

The improved dispersion of graphene in PVC nanocomposites, due to the *Curcuma longa* L. rhizome extract, was also confirmed in our previous studies. In [65], structure analysis by Raman spectroscopy showed better dispersion of the filler, which was presented in the nanocomposites in the form of few-layer sheets.

### 2.3. Thermal Properties of PVC/GN Nanocomposites

**Figure 5:**
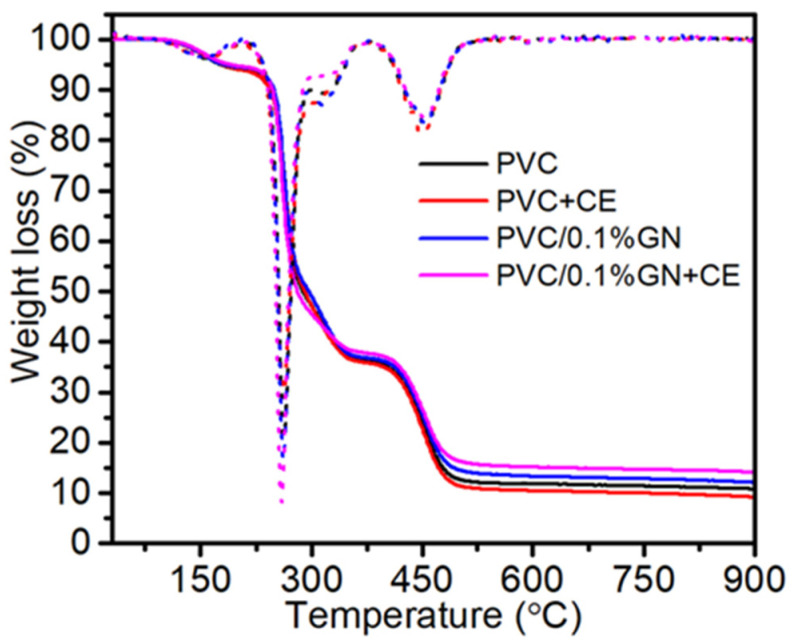
TGA thermograms of PVC, PVC + CE and PVC/0.1% GN, and PVC/0.1% GN + CE nanocomposites.

Next, thermogravimetric analysis was performed. Figure 5 presents TGA curves of a nanocomposite containing 0.1 wt.% of GN and PVC with the addition of CE and, for comparison, unmodified poly(vinyl chloride). The thermograms show mass loss at a temperatures up to 170 °C, which is related to the evaporation of residual THF [29,66]. The residual solvent in the films, regardless of their composition, was also found on the basis of the Raman spectrum [65]; its content was about 5.7% (Table 1) despite the applied evaporation and drying methods. This temperature range was proposed based on an earlier study, where a higher temperature would lead to the degradation of the material [67]. 

Therefore, the thermal degradation of poly(vinyl chloride) and nanocomposites is divided into two steps and is related to the decomposition of poly(vinyl chloride). The first step (from 200 to 375 °C) is related to the dehydrochlorination of the polymer matrix and the formation of a conjugated polyene structure. The second major mass loss in the range of 375 to 600 °C corresponds to thermal cracking of the carbonaceous conjugated polyene sequences and the formation of residual chars [22,29,66]. Based on the residual differential thermogravimetry (DTG) [24,68], the first and second decomposition steps were used to analyze the thermal stability of the obtained material, and the residual mass after the nanocomposite heating process was assessed. The results are summarized in Table 1 (standard deviation of the obtained mean values in brackets). The conducted thermogravimetric analysis proved a lack of statistically significant changes in the thermal stability of the obtained nanocomposite materials compared with unmodified PVC. The addition of CE also did not affect the thermal stability of PVC/GN nanocomposites.

Poly(vinyl chloride) is sensitive to heat, so it can undergo thermomechanical degradation processes under processing due to the progressive dehydrochlorination that can take place according to various mechanisms [16]. The determination of the thermal stability of PVC is extremely important due to the correct selection of processing parameters. A commonly used method for assessing thermal stability is the Congo red test, which was applied to determine the thermal stability time of the obtained nanocomposites. The results presented in Table 1 show that the introduction of graphene at an amount above 0.5 wt.% results in a slight improvement in the thermal stability of materials with no CE content. On the other hand, a small concentration of graphene in the PVC matrix accelerates the dehydrochlorination process. The authors [68] indicated that the lower stability of PVC/GN composites may be due to the fact that GN nanoflakes act as a reinforcing particulate filler that attracts Cl. The addition of *Curcuma longa* L. extract significantly extended the thermal stability time of poly(vinyl chloride) nanocomposites containing graphene, which is similar, regardless of the GN content. The improvement in thermal stability was attributed to the antioxidant properties of curcuminoids containing the phenolic -OH group effectively neutralizing free radicals [69]. The mechanism preventing the propagation of HCl release from PVC, thanks to the use of antioxidants, is applied to improve its thermal stability [70].

### 2.4. Swelling Behavior of PVC/GN Nanocomposites

Numerous studies confirm the chemical resistance of poly(vinyl chloride) against various types of compounds [22,71,72]. However, PVC may swell or even dissolve if contacted with ketones, ethers, and aromatic or chlorinated hydrocarbons [39]. PVC dissolves completely in THF and cyclohexanone, and it undergoes limited swelling when contacted with acetone. 

**Figure 6:**
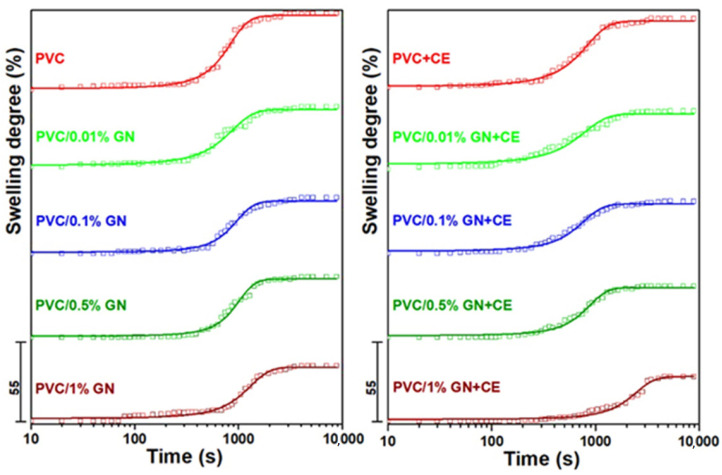
Swelling degree of PVC and PVC/GN nanocomposites vs. time.

Therefore, in this work, the chemical resistance of the proposed PVC/GN nanocomposites was tested by analyzing the swelling process in acetone. Swelling curves, that is, the dependence of the swelling degree as a function of time, are shown in Figure 6. 

On the basis of the obtained results, it was found that all the obtained materials undergo limited swelling, while the dependence of the swelling degree on the exposure time to the swelling agent has a shape of a sigmoid function. Therefore, Equation (1) was used to approximate the swelling curves [73]:

(1) $$S_{d}=\frac{S_{E}}{1+10^{\left(t_{M}-t\right)p}}$$

where

S_d_—swelling degree, %;
S_E_—equilibrium swelling, upper asymptote, %;
t_M_—time in which the swelling occurs with a maximum rate, s;
t—time of exposure to the swelling agent, s;
p—comparison parameter, s^−1^.

The parameters of the equation and the coefficient of determination R2 describing the degree of matching of the experimental results to the assumed model are summarized in Table 2. The proposed model describes the experimental results with high accuracy, as evidenced by the high values of the coefficient of determination (close to one).

It was found that the addition of CE to poly(vinyl chloride) reduces the equilibrium swelling degree (S_E_) by 8.8%. Materials with graphene, even at 0.01 wt.% of GN content, are characterized by a much lower S_E_ in comparison with PVC and PVC + CE. The values of the equilibrium swelling degree decreased, thanks to the use of GN in nanocomposites. PVC/1% GN + CE was determined by the lowest S_E_, which means an improvement by 43.5% in comparison with PVC and by 38.1% in comparison with PVC + CE. It should also be emphasized that nanocomposites with *Curcuma longa* L. extract are characterized by a lower value of the discussed parameter compared with the corresponding materials without CE. This was attributed to the improved dispersion of the filler in the polymer matrix, which was confirmed by significantly lower values of the equilibrium swelling degree for materials with 0.5 and 1 wt.% of GN. As the graphene content of the nanocomposites increased, the t_M_ value was also increased, which confirmed the chemical resistance of nanocomposites to acetone. A similar effect can be achieved by additional chlorination of the polymer chain [73]; however, the use of graphene is an environmentally friendly solution, and it does not require a chemical change in the structure of poly(vinyl chloride). In the literature, it has been indicated that the increased chemical resistance of PVC, thanks to the use of carbon fillers, is the result of increasing its stiffness and reducing its free volume. As a consequence, this leads to less solvent access to the polymer chain [71]. In the case of the discussed materials, this mechanism is very likely, which can be explained by the improvement of the graphene dispersion in the polymer matrix compared with bare GN.

### 2.5. Dynamic Mechanical Thermal Analysis of PVC/GN Nanocomposites

**Figure 7:**
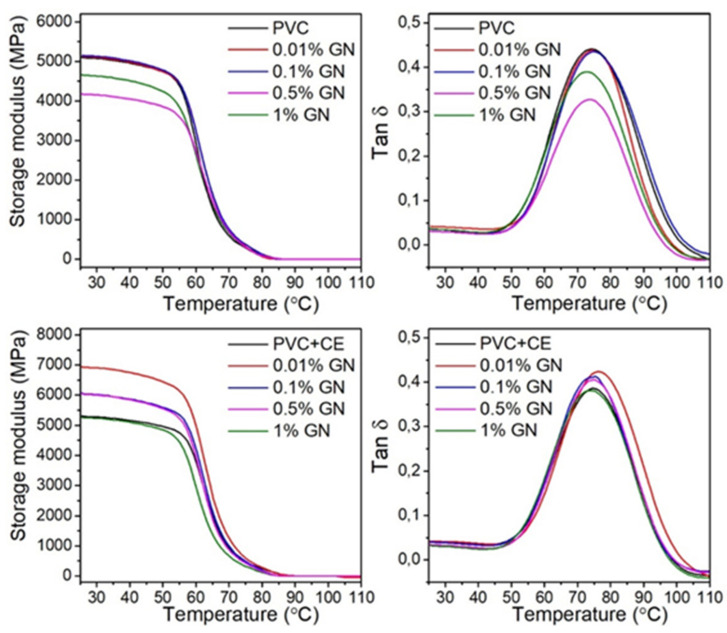
Storage modulus and tan δ of PVC and PVC/GN nanocomposites.

In order to assess the influence of GN on the mobility of PVC macromolecule segments, the dynamic mechanical properties of nanocomposites were investigated using tensile testing. Figure 7 shows the changes in the storage modulus (E’) and the loss coefficient (Tan δ) of the obtained materials as a function of temperature.

On the basis of the obtained results, it was observed that the value of the storage modulus decreased with the increase in temperature for all nanocomposites, regardless of their composition, which resulted from the increase in the mobility of the polymer chain segments [26]. The E’ values of composites with no CE content decrease with the increase in the filler content in PVC, which may be related to the crumpled morphology of the applied GN and, above all, to the heterogeneous dispersion of the filler in the matrix [25,40]. The use of the extract resulted in the increase in the storage modulus of nanocomposites in the temperature range below the glass transition temperature compared with both PVC and composites with no CE content. Additionally, for samples containing up to 0.5 wt.% of GN in the polymer matrix, an increase in E’ in the matrix is observed. The increase in E’ indicates strong interfacial interactions between GN and PVC because of the use of CE, as well as the limitation of the mobility of polymer chain segments by a well-dispersed filler and, thus, an increase in the stiffness of nanocomposites [26,31,74].

The values of the glass transition temperature of nanocomposites and PVC as their matrices, determined on the basis of the dependence of Tan δ as a function of temperature, are summarized in Table 1. Extract from *Curcuma longa* L. had practically no effect on the T_g_ value of PVC with no filler. However, its application influenced the increase in T_g_ of PVC/GN + CE nanocomposites compared with both PVC and PVC/GN, while the highest value of this temperature was observed in the case of the PVC/0.01% GN + CE sample (increase by 2 °C compared with the T_g_ value of the matrix material). An increase in the concentration of graphene to 0.1 and 0.5 wt.% increases the T_g_ value about 1 °C what showing similar values to unmodified material. Therefore, it can be concluded that the amount of applied CE (1 wt.% based on PVC weight) is insufficient to increase filler–polymer interactions at higher GN contents.

In the composite without CE, no statistically significant effect of graphene at an amount up to 0.1 wt.% on the glass transition temperature of PVC was found. An increase in the content of graphene to 0.5 and 1 wt.% caused its decrease by 1 °C compared with the T_g_ value of unmodified PVC.

An increase in temperature associated with the maximum Tan δ value and an increase in the storage modulus in CE-containing materials indicate that well-dispersed graphene affects molecular dynamics, limiting the segmental movement of PVC polymer chains in nanocomposites. This leads to an increase in the glass transition temperature compared with unmodified PVC films.

### 2.6. Mechanical Properties of PVC/GN Nanocomposites

**Figure 8:**
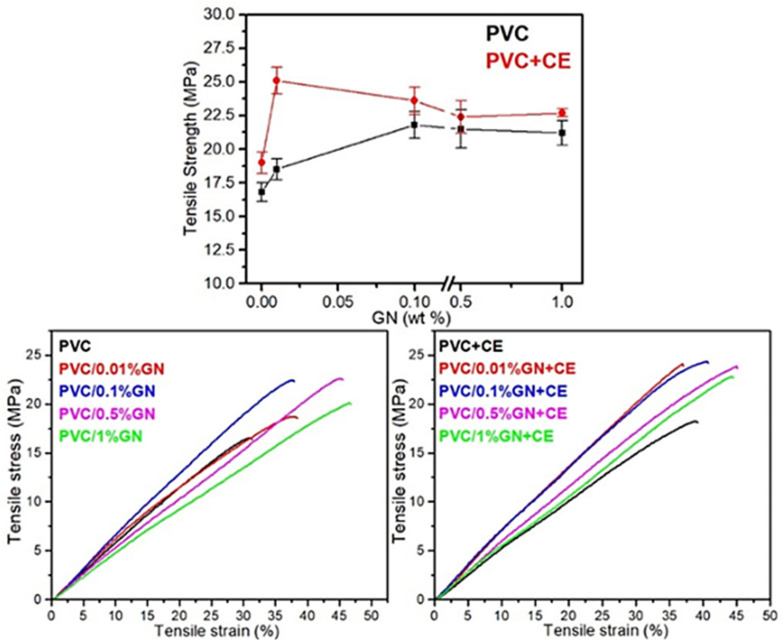
Mechanical properties of PVC and PVC/GN nanocomposites.

Figure 8 shows exemplary stress–strain curves of PVC and nanocomposites on the matrix and the determined tensile strength (TS). The results of the tests of mechanical properties are summarized in Table S1, included in the Supplementary Materials. The course of the stress–strain curve shows no yield point regardless of the material composition, which is as expected. In the case of the film with no CE addition, an increase in the slope of the stress–strain curve can be observed, compared with the slope of the unmodified PVC curve. This is only in the case of the PVC/0.1% GN sample. The slope of the other curves is smaller. 

The slope of all stress–strain curves of a PVC/GN + CE foil is greater than the slope of the PVC + CE sample curve. A significant (and the largest) increase in the stress–strain curve slope was first observed for the sample containing as little as 0.01 wt.% of GN, indicating a higher stiffness of the materials, and improved the dispersion of the filler. 

The presence of graphene flakes in the PVC matrix reduces the mobility of polymer chains, which leads to an increase in the stiffness of the material [64]. As the interfacial interactions in PVC/GN composites are of physical nature and may be insufficient to increase the stiffness of polymer chains at higher filler contents [25], the slope in the stress–strain curves decreases for PVC/0.5% GN and PVC/1% GN samples (Figure 8). The addition of CE caused an increase in interfacial interactions between the matrix and a nanofiller, as evidenced by the increase in stiffness of nanocomposites compared with the composites without CE. 

Regardless of the composition of nanocomposites, the introduction of graphene into the PVC matrix resulted in an increase in tensile strength. The TS value of PVC with no dispersant was 16.8 MPa, and the addition of CE slightly increased this value (up to 19 MPa). The highest increase in tensile strength was observed for the samples of PVC/0.1% GN (by 29.8% as compared with PVC) and PVC/0.01% GN + CE (by 49.4% as compared with PVC and by 32.1% as compared with PVC + CE). A further increase in the content of graphene in nanocomposites, regardless of the presence of the dispersant, did not cause statistically significant changes in tensile strength, and the obtained mean values indicated a gradual decrease in TS. In [25,64,75], the authors indicated the presence of the percolation threshold, beyond which no significant increase in mechanical properties or even their deterioration was observed. This fact results from the secondary agglomeration of graphene and the introduction of defects in the structure of nanocomposites along with the increasing content of the filler, for example, air bubbles sticking in the matrix. The presented results demonstrate the beneficial use of the extract from *Curcuma longa* L. as a graphene stabilizer in the PVC matrix, improving the mechanical properties of the nanocomposite. This is confirmed by an almost 35.7% increase in TS of a PVC/0.01% GN + CE film as compared with the material with the same content of graphene without CE. In the paper [65], it was indicated that the stabilization of the GN (using CE) dispersion in solutions and the poly(vinyl chloride) matrix occurs as a result of π–π interactions. As demonstrated in [35,38], intermolecular interactions can be successfully used to improve the filler–polymer interactions in PVC/GN nanocomposites. This effect was observed in the present research, but the constant amount of the applied dispersant (1 wt.%) was insufficient to increase the above-mentioned interactions at higher GN contents. This is evidenced by the lack of a statistically significant difference in the tensile strength values of nanocomposites with and without the addition of CE, containing 0.1 and 0.5 wt.% of graphene, respectively, and similar tensile strength values of films containing 1 wt.% of GN. In studies [25,31], it was pointed out that the use of additional nanocomposite components requires their correlation with the content of graphene. 

To summarize, it can be clearly stated that the improvement of the mechanical properties of PVC/GN nanocomposites depends on the homogeneous dispersion of graphene in the polymer matrix. However, on the other hand, the dense GN network limits the penetration of poly(vinyl chloride) macromolecules between its layers, which makes the matrix discontinuous [25,30,64]. This explains the lack of a significant improvement in the mechanical properties of materials with significantly improved filler dispersion, for example, PVC/1% GN + CE. The filler–polymer interaction also has an impact on the limited improvement in these properties. The lack of oxygen-containing functional groups (the applied filler contained only 2.5% of oxygen—manufacturer’s data) on the GN surface results in its high integrity and good mechanical properties, which directly determines the improvement of the mechanical properties of PVC/GN nanocomposites. However, the interfacial interactions in materials containing such fillers have the nature of physical bonds, too weak to improve tensile strength at high graphene contents in the composite [59,64,75]. As shown in the present study, the use of CE as a dispersant can improve interfacial interactions in nanocomposites. However, further research is needed to determine the appropriate extract content in relation to GN, which affects both the improvement of filler dispersion and the increase in filler–polymer interaction.

### 2.7. Electrical Properties of PVC/GN Nanocomposites

**Figure 9:**
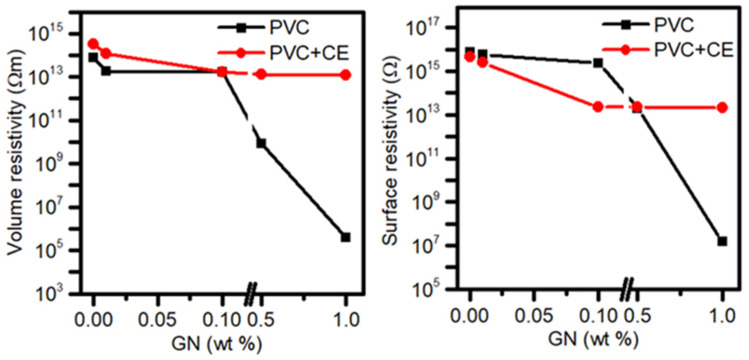
Surface and volume resistivity of PVC and nanocomposites.

Literature reports [25,31,67] present that it is possible to modify the electrical properties of poly(vinyl chloride) with the use of graphene. The influence of GN on the resistivity or conductivity of PVC/GN nanocomposites is significantly related to the dispersion of the filler in the polymer matrix. Figure 9 shows the results of the resistivity tests of the obtained materials, which are summarized in Table S1 (Supplementary File).

The surface resistivity of poly(vinyl chloride) was determined to be 7.5 × 10^15^, while in the case of PVC + CE, it equaled 4.5 × 10^15^ Ω. The surface resistivity value decreases slightly for the nanocomposite without CE with the increase in graphene content to 0.1 wt.%, and its significant change occurs only when the filler concentration in the composites is 0.5 and 1 wt.%. The lowest resistivity, which equals 1.5 × 10^7^ Ω, is characteristic for the PVC/1% GN sample. This value allows the composite to be classified as an antistatic material [31,32]. Nanocomposites containing CE did not show such a sharp decrease in surface resistivity; after the decrease in its value to 1.7 × 10^13^ for the PVC/0.1% GN + CE sample, no further significant changes were observed. The volume resistivity of PVC/GN composites with no CE also decreases with the increase in graphene concentration in the matrix, and its step change occurs at 0.5 wt.% of the filler. The materials containing 1 wt.% of GN were characterized by the lowest resistivity, and it equaled 3.9 × 10^5^ Ω m, which means a change by nine orders of magnitude compared with PVC (7.7 × 10^14^). Considering the significant agglomeration of graphene, such a large change in resistivity is, on the one hand, a surprising effect, and on the other hand, it indicates that the GN dispersion was sufficient to create conduction paths. PVC/GN + CE composites, as in the case of surface resistivity, did not show large changes in volume resistivity as the filler content in the matrix increased. 

No change in the resistivity of CE-containing materials, despite a significant improvement in GN dispersion, results from the π–π interactions between graphene and curcuminoids. Some studies show the disturbances in the displacement of π electrons on the graphene surface, leading to the significant deterioration of its electrical properties [35,67]. Nevertheless, the application of CE improves the interfacial interactions, leading to the well dispersion of GN in the polymer matrix.

## 3. Experimental Section

### 3.1. Materials

Graphene-based nanopowder with a flake thickness of 1.6 nm (maximum of 3 atomic monolayers), a flake length of 10 µm, and a specific surface area of 400 ÷ 800 m^2^g^−1^ was purchased from USA Graphene Laboratories Inc. Graphene was dispersed in a poly(vinyl chloride) solution using unmodified suspensive poly(vinyl chloride) Neralit 601 (Czech Republic, Spolana s.r.o. Anwil S.A. group) with a K number of 59–61, a bulk density of 0.56–0.63 g cm^−3^, a specific density of 1.39 g cm^−3^, and 97% purity. As a solvent for PVC, tetrahydrofuran (THF) (Chempur, Piekary Śląskie, Poland) was used. *Curcuma longa* L. rhizome extract was used as a stabilizer of graphene in a PVC solution. Materials for the analysis of the extract with HPLC grade of methanol, acetonitrile, and water were purchased from Sigma-Aldrich (St. Louis, MO, USA). Acetic acid (99%), curcumin (99.5%), demethoxycurcumin (≥98), and bisdemethoxycurcumin (99%) were supplied from Sigma-Aldrich (USA).

### 3.2. Preparation of Curcuma longa L. Rhizome Extract

*Curcuma longa* L. extract was prepared by extraction in a B-811 Büchi Soxhlet extractor (Byrne, Switzerland), where 20 g of *Curcuma longa* L. rhizome powder (Heuschen & Schrouff, Landgraaf, The Netherlands) was placed in a glass thimble and extracted with methanol (Chempur, Poland). Extraction was performed in 80 °C for 3 h. Then, the solvent was evaporated from the extract at 60 °C for 24 h, pure extract. The methodology and analysis of the obtained extract are presented in the Supplementary Materials.

### 3.3. Preparation of PVC/GN Nanocomposites

In the first stage of preparation of the nanocomposites, poly(vinyl chloride) was dissolved in THF at 25 °C for ca. 48 h, yielding a solution with a concentration of 3 wt.% Next, graphene was added to the solution and dispersed for 60 min at 20 °C. The dispersion of graphene flakes was enhanced ultrasonically with a frequency of 20 kHz and 40% amplitude, using a SONOPULS rod-shaped probe homogenizer from Bandelin. The suspension containing curcuma extract was prepared in the same way, adding 1% of CE to the PVC weight. The amount of graphene in the prepared dispersions was 0.01%, 0.1%, 0.5%, and 1% per the polymer weight.

Then, the thin films of PVC/GN nanocomposites were obtained by the solvent evaporation method onto Petri dishes having a diameter of 7 cm, where the solvent was evaporated at 50 °C for 24 h. To remove the THF residue, the film was dried in a vacuum drier under reduced pressure (max 20 mbar absolute) at 50 °C for 2 weeks. Films of unmodified PVC and PVC with 1 wt.% of CE were obtained under the same conditions. All samples were coded to take into account the graphene content and the presence of curcumin, for example, a sample containing only GN at a concentration of 0.01 wt.% as PVC/0.01% GN versus a sample containing the same graphene content and turmeric extract PVC/0.01% GN + CE.

### 3.4. Methods of Curcuma longa L. Rhizome Extract Analysis

The identification of *Curcuma longa* L. rhizome extract was carried out by Fourier-transform infrared spectroscopy (FTIR) using the ATR technique. The study was performed with an Alpha apparatus from Bruker in the range of 400–4000 cm^−1^. A total of 32 scans at a resolution of 4 cm^−1^ were performed.

For HPLC analysis, the fresh turmeric extract for chromatographic determination was dissolved in methanol. The methanol solution was filtered through a 0.45 µm Acrodisc 13 mm nylon filter (Gelman, St. Louis, MO, USA). HPLC analysis was performed using a Shimadzu UFLCXR with a SPD-M30A photodiode array detector. The UV–vis spectra were taken in the range of 200–800 nm. A Phenomenex Luna 3u C18(2) 100A (150 × 3.0 mm) column was used. The column temperature was set at 40 °C. The profile of the gradient elution was: (A) water (0.25% HOAc) and (B) acetonitrile l, 0–17 min, 40–60% B; 17–28 min, 60–100% B; 28–35 min, 100% B; 35–40 min, 100–40% B, at a flow rate of 0.8 mL min^−1^.

For the quantitative analysis of curcumin, demethoxycurcumin, bisdemethoxycurcumin, the following concentrations of each compound were prepared: 2, 10, 20, 40, and 80 µg mL. A calibration curve and a correlation coefficient (R^2^) were determined for each compound. The limit of detection (LOD) was defined as the signal-to-noise (S/N) ratio of 3. The limit of quantification (LOQ) was defined as S/N = 10. Concentrations of curcumin, demethoxycurcumin, and bisdemethoxycurcumin in the extract were calculated based on the regression equations.

The curcumin extract was also analyzed on an Agilent 7890B GC System gas chromatograph with an Agilent 5977B GC/MSD mass spectrometry detector with an HP-5MS column (0.25 mm × 30 m × 0.25 µm). The analyses were performed under the following chromatographic conditions: injector temperature of 250 °C, detector temperature of 280 °C, oven temperature program from 50 °C/4 min increase of 15 °C min^−1^ to 300 °C (maintained for 10 min). Helium was used as a carrier gas, flow 1 mLmin^−1^. The volume of the sample was 1 μL. The substances were identified by comparing the obtained MS spectra with the spectra of the NIST17.L Mass Spectrum Library.

### 3.5. Characterization of PVC/GN Nanocomposites

The structure of the prepared nanocomposites was studied using a ZEISS EVO 40 scanning electron microscope (SEM). The samples for SEM observation were fractured cryogenically and sputtered with gold nanometric layer.

The filler dispersion in the matrix was also determined by a Nanosurf atomic force microscope (AFM) (Liestal, Switzerland) in contact mode using a PPP-XYCONTR measuring probe (NANOSENSORS, Neuchatel, Switzerland), where the fillet radius of the aluminum-coated blade was 7 nm, while its line inclination angle was 20 degrees. The surface roughness was measured over a distance of 50 µm by collecting data from 50 measurement lines at 1 nm intervals. The number of measuring points per one line was 5000. The sample scan rate during surface evaluation by roughness measurement was 12.5 µm s^−1^. Three-dimensional images were made within an area of 10 × 10 µm. In order to determine the surface topography, 500 lines were measured in the given area (at 20 nm intervals), and the number of measuring points per one line was 5000. The sample scan rate was 5 µm s^−1^.

The thermal stability of the nanocomposites was assessed by the thermogravimetric method (TGA) using a TG 209 F3 Tarsus apparatus (Netzsch). The heating rate was about 10 °C min^−1^ in an open ceramic crucible under a nitrogen atmosphere in the temperature range of 30 to 900 °C. The change in sample mass as a function of temperature was measured. Static thermal stability tests using the Congo red test at 200 °C measuring the thermal stability time, that is, the time when the sample shows no signs of destruction in the form of hydrogen chloride release and color changes of the indicator paper, were also performed [76].

The resistance of the obtained materials to swelling in acetone was tested in accordance with the method proposed in our earlier study [67]. The measurement consisted in determining the change in swelling degree (S_d_) (see Equation (2)), depending on the immersion time in the swelling agent. The changes in the sample diameters were determined on the basis of photos using the NIS-Elements 4.0 software. The frequency of taking pictures depended on the exposure time to the swelling agent. The measurement temperature was 20 °C, and the initial diameter of the samples was h_0_ = 10 mm,

(2) $$S_{d}=\frac{h-h_{0}}{h_{0}}\cdot 100\%$$

where

h—sample diameter after time t (mm);
h_0_—initial sample diameter (mm).

Next, the thermomechanical and mechanical properties of nanocomposites were investigated. Thermal analysis of dynamic mechanical properties was performed on a DMA Artemis device (Netzsch Group, Selb, Germany). The values of the storage modulus (E’) and the loss angle tangent (tanδ) as a function of temperature were determined. The position of the maximum tanδ was assumed to be the glass transition temperature. The test was performed in the tensile mode (measuring length of 10 mm, sample width of 5 mm, thickness of 0.18 ± 0.02 mm) with a deformation of 10 μm in the temperature range of 25–110 °C and with a heating rate of 2 °C min^−1^. The deformation was determined with a frequency of 1 Hz. A relatively small amplitude and low frequency of deformation allow for measurement in the linear viscoelastic range [77,78].

A study of tensile properties was carried out with the use of an in-house built tensile tester equipped with a Zemic H3-C3-25 kg-3B strain gauge beam characterized by a measuring range of 25 kg and a maximum measurement error of 0.02%. The tests were carried out on samples with a thickness of 0.18 ± 0.02 mm and a width of 2 ± 0.1 mm, while the length of the measuring section L_0_ was 1.5 mm. The materials were stretched at a constant rate of 0.1 mm s^−1^, and the mechanical properties of the nanocomposites were determined on the basis of the measurement data. The force from the strain gauge beam was recorded with an accuracy of 0.001 N every 0.014 s [79,80]. 

Electrical properties of nanocomposites were determined by examining surface and volume resistivity. The measurement was performed with a measuring system consisting of a 6517A electrometer and a 8009 measuring chamber (Keithley Instrument Inc., Cleveland, OH, USA). Measurements of volume and surface resistivity were carried out on film samples with a diameter of 70 mm in air at a temperature of 23 °C and humidity of 50% at a voltage of 10 V. 

In order to analyze the obtained results, the Origin 8.6 Pro software with implemented statistical analysis modules was used. ANOVA with Tukey’s post hoc test was used to compare the significance of the difference for the mean values of the obtained results. The normal distribution was confirmed by the Shapiro–Wilk test, and the homogeneity of variance by Levene’s test. All analyses were performed assuming a significance level below 0.05.

## 4. Conclusions

In this work, a nanocomposite based on poly(vinyl chloride) and graphene stabilized with *Curcuma longa* L. extract was proposed. The use of the environmentally friendly plant extract significantly improved the dispersion of GN in the PVC matrix, which was confirmed by SEM and AFM. CE did not affect the thermal stability of PVC measured by the TGA method, but improved the thermal stability time (determined by means of the Congo red test) due to the antioxidant properties of this dispersant. The enhancement of the stability time indicates promising potential for its application for the production of nanocomposites by the melt mixing method. Moreover, a significant improvement in mechanical and thermomechanical properties at a very low content of GN (0.01 wt.%) is noteworthy, which indicates an increase in the filler–polymer interfacial interactions resulting from the use of CE. The use of the extract causes a significant deterioration of the electrical properties of graphene, resulting in the lack of changes in the resistivity of nanocomposites.

*Curcuma longa* L. extract has great potential to be used as a dispersant in PVC/GN nanocomposites, mainly because of its lack of negative impact on health and the environment. However, its application requires further research aimed at developing an optimal composition of certain composites in which the amount of CE will be correlated with the content of GN.

## Figures and Tables

**Table 1:** Thermal properties.

Material	Cont. of THF, %	Max. DTG I, °C	Max. DTG II, °C	Residual Mass, %	Congo Red Test, Min	T_g_, °C
PVC	5.3 (0.9)	268.4 (5.5)	456.5 (7.2)	8.7 (0.4)	3.0 (0.3)	74.3 (0.04)
PVC + CE	5.8 (0.2)	267.4 (4.9)	455.6 (8.1)	8.6 (1.2)	5.1 (0.8)	74.4 (0.2)
PVC/0.01% GN	5.9 (0.1)	268.2 (5.0)	459.0 (5.5)	9.5 (1.1)	2.5 (0.5)	74.5 (0.4)
PVC/0.01% GN + CE	5.8 (0.5)	264.3 (6.7)	456.1 (6.6)	11.0 (2.9)	5.7 (0.1)	76.3 (0.2)
PVC/0.1% GN	5.7 (0.2)	268.2 (5.7)	461.6 (4.2)	10.8 (1.2)	2.2 (0.2)	74.6 (0.2)
PVC/0.1% GN + CE	5.6 (0.3)	268.7 (8.6)	461.1 (3.6)	11.0 (2.8)	6.9 (0.3)	75.4 (0.1)
PVC/0.5% GN	5.8 (0.5)	267.5 (3.3)	460.4 (4.7)	10.8 (1.4)	4.0 (0.5)	73.3 (0.3)
PVC/0.5% GN + CE	5.7 (0.5)	263.4 (4.0)	459.5 (5.4)	11.9 (2.1)	5.7 (0.6)	75.3 (0.3)
PVC/1% GN	5.9 (0.9)	267.6 (7.1)	457.6 (7.8)	11.5 (1.8)	3.9 (0.2)	73.3 (0.2)
PVC/1% GN + CE	5.6 (0.3)	269.0 (6.1)	459.8 (6.6)	12.7 (0.9)	5.5 (0.2)	74.4 (0.3)

**Table 2:** Parameters of the model describing the swelling process.

Material	S_E_, %	t_M_, s	p, s^−1^	R^2^
PVC	53.3 (0.5)	725 (10)	0.002 (0.00007)	0.994
PVC + CE	48.6 (0.4)	672 (10)	0.0017 (0.00005)	0.995
PVC/0.01% GN	41.4 (0.6)	702 (19)	0.0016 (0.00008)	0.986
PVC/0.01% GN + CE	38.4 (0.7)	599 (24)	0.0016 (0.0001)	0.974
PVC/0.1% GN	36.3 (0.4)	859 (16)	0.0019 (0.00009)	0.990
PVC/0.1% GN + CE	34.2 (0.4)	643 (14)	0.002 (0.00009)	0.990
PVC/0.5% GN	40.4 (0.5)	903 (16)	0.0019 (0.0001)	0.991
PVC/0.5% GN + CE	34.4 (0.4)	753 (14)	0.0019 (0.00008)	0.991
PVC/1% GN	37.4 (0.5)	1086 (24)	0.0012 (0.00005)	0.988
PVC/1% GN + CE	30.1 (0.7)	2091 (67)	0.0006 (0.00004)	0.979

## Data Availability

The data that support the findings of this study are available in the Supplementary Materials of this article or from the corresponding author upon reasonable request.

## Supplementary Materials

The following supporting information can be downloaded at: https://www.mdpi.com/article/10.3390/molecules27228081/s1, Figure S1: Mass spectrum of Ar-curcumene; Figure S2: Mass spectrum of (-)-zingiberene; Figure S3: Mass spectrum of β-sesquiphellandrene; Figure S4: Mass spectrum of Ar-turmerone; Figure S5: Mass spectrum of α-turmerone; Figure S6: Mass spectrum of β-turmerone; Figure S7: Mass spectrum of (6R,7R)-bisabolene; Figure S8: Mass spectrum of (E)-atlantone; Table S1: Mechanical and electrical properties of PVC/GN nanocomposites.

## Sample Availability

Samples of the compounds are not available from the authors.
