# Supplementary material for: Curcuma longa L. Rhizome Extract as a Poly(vinyl chloride)/Graphene Nanocomposite Green Modifier
Source: Molecules. 2022 Nov 21;27(22):8081. doi: 10.3390/molecules27228081 (PMC9698460; doi:10.3390/molecules27228081)
Supplement: Supplementary file 1 [file molecules-27-08081-s001.zip › molecules-2029704-supplementary.pdf]

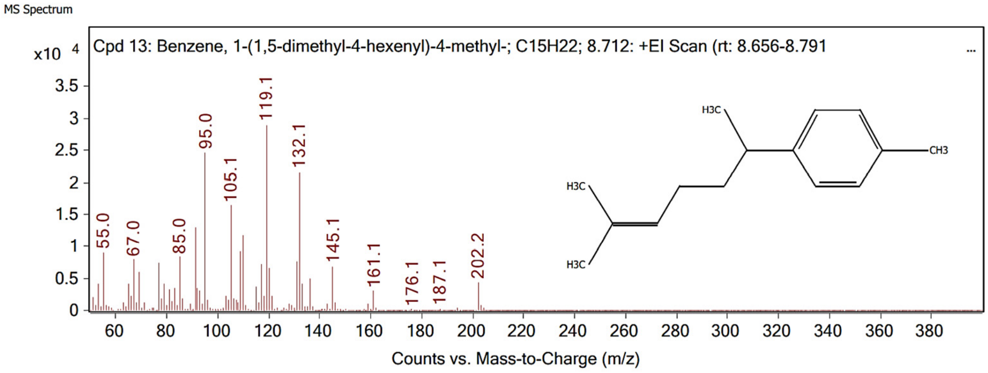

Figure S1. Mass spectrum of Ar-Curcumene

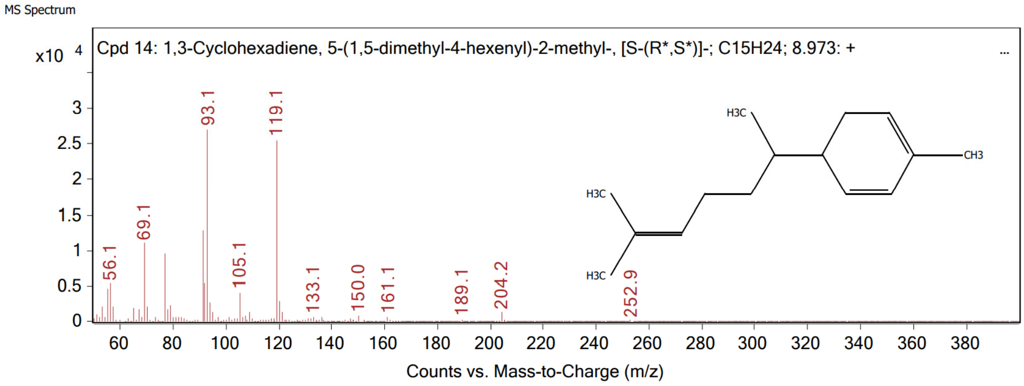

Figure S2. Mass spectrum of (-)-Zingiberene

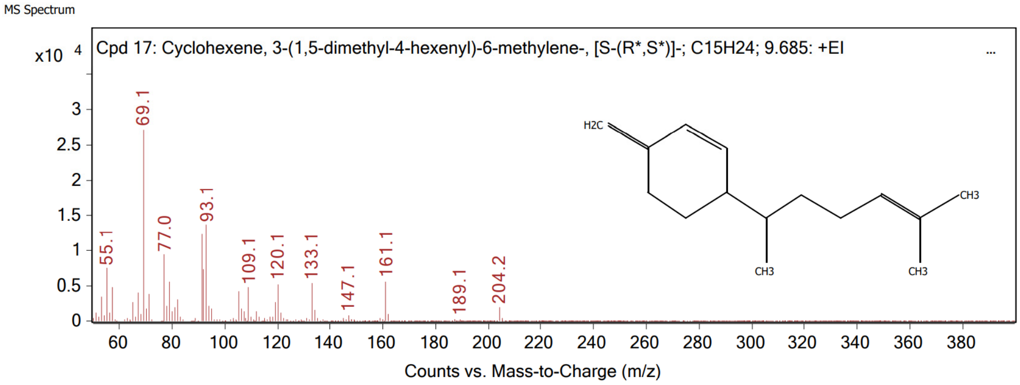

Figure S3. Mass spectrum of β-Sesquiphelandrene

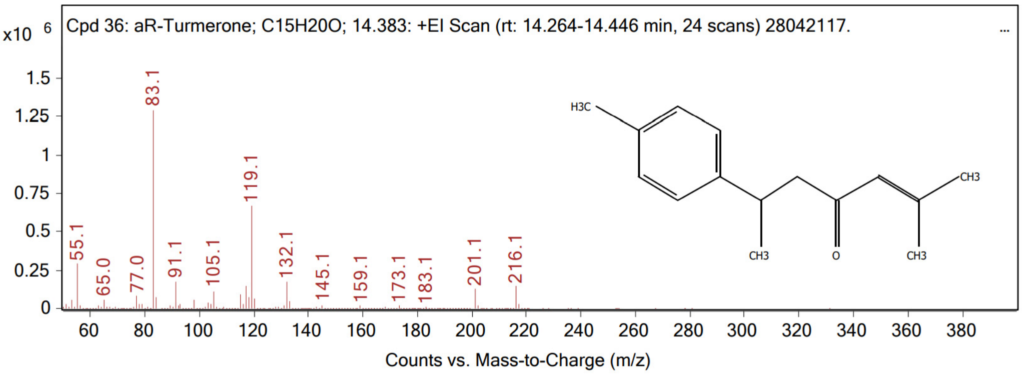

Figure S4. Mass spectrum of Ar-turmerone

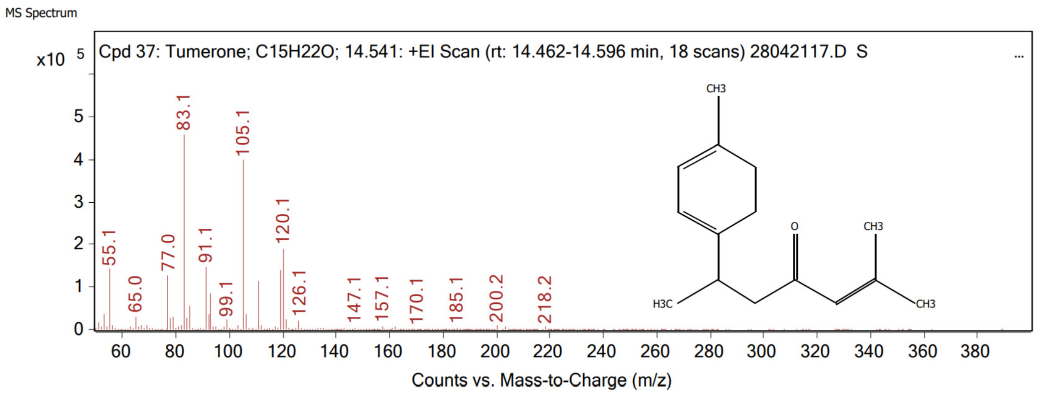

Figure S5. Mass spectrum of α-turmerone

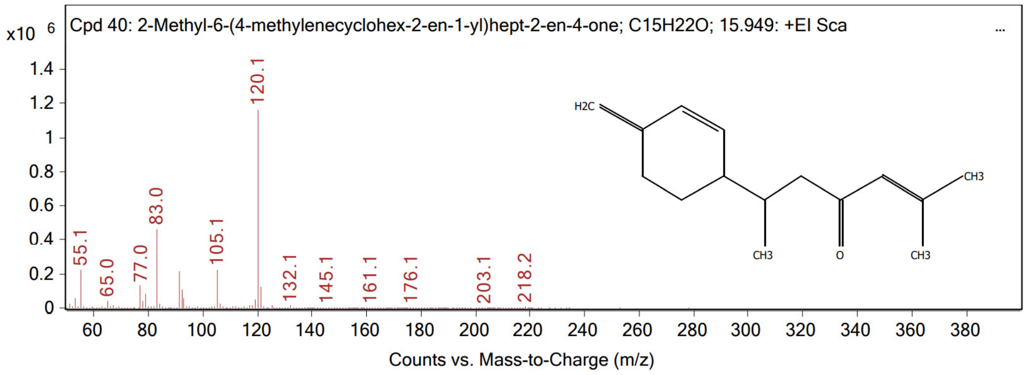

Figure S6. Mass spectrum of β-turmerone

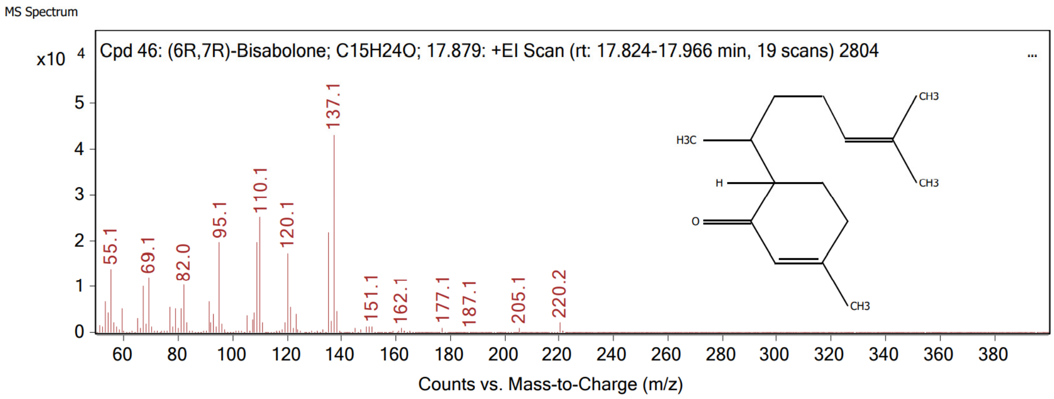

Figure S7. Mass spectrum of (6R,7R)-Bisabolene

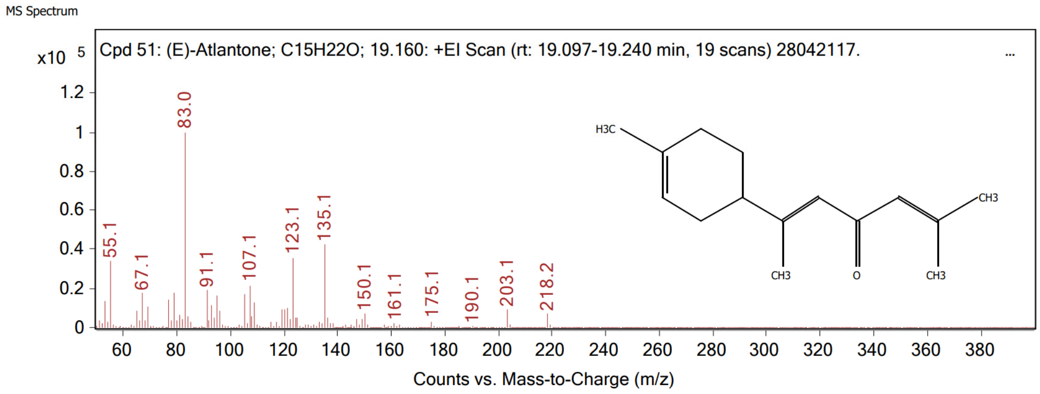

Figure S8. Mass spectrum of (E)-atlantone

Table S1. Mechanical and electrical properties of PVC/GN nanocomposites

Material	Tensile strenght, MPa	Volume resistivity, Ωm	Surface resistivity, Ω
PVC	16.8 (0.7)	7.7×10^{14} (3.8×10^{11})	7.5×10^{15} (6.7×10^{13})
PVC+CE	19.0 (0.8)	3.1×10^{14} (2.2×10^{11})	4.5×10^{15} (3.9×10^{13})
PVC/0.01%GN	18.5 (0.8)	1.9×10^{13} (1.5×10^{11})	5.8×10^{15} (6.9×10^{13})
PVC/0.01%GN+CE	25.1 (1.0)	1.2×10^{14} (8.2×10^{11})	2.5×10^{15} (1.9×10^{13})
PVC/0.1%GN	21.8 (1.0)	1.6×10^{13} (9.8×10^{10})	2.4×10^{15} (1.9×10^{13})
PVC/0.1%GN+CE	23.6 (1.0)	1.7×10^{13} (1.7×10^{11})	2.3×10^{13} (2.7×10^{11})
PVC/0.5%GN	21.5 (1.4)	8.4×10^{9} (8.4×10^{7})	2.0×10^{13} (1.8×10^{11})
PVC/0.5%GN+CE	22.4 (1.2)	1.2×10^{13} (1.2×10^{11})	2.2×10^{13} (4.2×10^{11})
PVC/1%GN	21.2 (0.9)	3.9×10^{5} (3.5×10^{4})	1.5×10^{7} (2.1×10^{5})
PVC/1%GN+CE	22.7 (0.3)	1.2×10^{13} (9.9×10^{10})	2.11×10^{13} (4.9×10^{11})
